# Carbon-Nanowall Microporous Layers for Proton Exchange Membrane Fuel Cell

**DOI:** 10.3390/membranes12111064

**Published:** 2022-10-29

**Authors:** Adriana Elena Balan, Bogdan Ionut Bita, Sorin Vizireanu, Gheorghe Dinescu, Ioan Stamatin, Alexandra Maria Isabel Trefilov

**Affiliations:** 1 Faculty of Physics, University of Bucharest, 077125 Bucharest-Măgurele, Romania; 2 National R&D Institute for Laser, Plasma and Radiation Physics (INFLPR), 077125 Bucharest-Măgurele, Romania

**Keywords:** carbon nanowalls, plasma, chemical vapor deposition, microporous layer, proton exchange membrane fuel cell

## Abstract

The cathode microporous layer (MPL), as one of the key components of the proton exchange membrane fuel cell (PEM-FC), requires specialized carbon materials to ensure the two-phase flow and interfacial effects. In this respect, we designed a novel MPL based on highly hydrophobic carbon nanowalls (CNW). Employing plasma-assisted chemical vapor deposition techniques directly on carbon paper, we produced high-quality microporous layers at a competitive yield-to-cost ratio with distinctive MPL properties: high porosity, good stability, considerable durability, high hydrophobicity, and substantial conductivity. The specific morphological and structural properties were determined by scanning electron microscopy, X-ray photoelectron spectroscopy, and Raman spectroscopy. Thermo-gravimetric analysis was employed to study the nanostructures’ thermal stability and contact angle measurements were performed on the CNW substrate to study the hydrophobic character. Platinum ink, serving as a fuel cell catalyst, was sprayed directly onto the MPLs and incorporated in the FC assembly by hot-pressing against a polymeric membrane to form the membrane-electrode assembly and gas diffusion layers. Single-fuel-cell testing, at moderate temperature and humidity, revealed improved power performance comparable to industrial quality membrane assemblies (500 mW cm^−2^ mg^−1^ of cathodic Pt load at 80 °C and 80% RH), with elevated working potential (0.99 V) and impeccable fuel crossover for a low-cost system.

## 1. Introduction

Proton exchange membrane fuel cells (PEMFCs) are considered a promising option for future zero-emission mobility, due to their high-power density enabled by the electrochemical reaction of hydrogen and oxygen [1,2,3]. However, the long-term operation of PEMFCs is affected by the stability of the component materials reflected in the time-decreasing efficiency of the membrane-electrode assembly (MEA). An MEA consists of a proton exchange membrane sandwiched between catalyst-supporting gas diffusion layers (GDLs) on the anode and cathode side [4]. For the proper and efficient functioning of the system, a fine balance of gas-liquid flows is required and GDL’s role in water management is crucial. It must maintain the hydration of the membrane for high proton conductivity, remove by-produced water from the catalyst layer to prevent the electrodes from flooding, conduct the gas flow during operation to the catalyst layer, and transfer heat during cell operation.

A typical GDL is composed of two layers: the microporous layer (MPL)—a carbon material mixed with a hydrophobic polymeric binder deposited on a second layer, which is a porous backing substrate with high electric conductivity, e.g., carbon paper. Mainly, the MPL helps to reduce mass transport limitations through improved water removal and an accompanying reduction in flooding, to minimize the contact resistance between the GDL and the catalyst layer, and to limit the loss of the catalyst to the GDL interior [5]. Currently, carbon black is widely used as the MPL. However, its erratic structure leads to poor stability and low electron transfer, and impedes gas-water management, making it inadequate for long-term fuel cell operation [6]. Extensive research has been dedicated to advanced carbonic materials with favorable properties: corrosion resistance, mechanical strength, high thermal and electrical conductivities, low fabrication costs, and environmental friendliness [6,7,8,9]. A wide range of materials such as carbon aerogels, xerogels, carbon nanotubes, and graphene have thus been explored [6,10,11,12,13].

In this context, we employ carbon nanowalls (CNWs)—inexpensive advanced nanostructured carbon materials, which have attracted increasing attention in recent years due to their properties and tailoring properties from the deposition conditions. They are carbon nanostructures formed of vertically-aligned multi-layered graphene domains with different interlayer spacing arranged vertically on a substrate [14,15]. Depending on the synthesis method, CNWs with specific properties can be obtained. It is already proven that high-quality CNWs can be obtained by plasma-enhanced chemical vapor deposition as the growth rate can be controlled through the discharge parameters of plasma [16,17,18,19]. Besides surface functionalization and defect density control, PECVD opens the possibility of the large-scale synthesis of advanced 2D carbon nanostructures [20]. Due to their high electric conductivity, good thermal stability, very large specific surface area, chemical stability, and physically robust shape owing to a high aspect ratio [21], several applications have been considered for CNWs, such as electrode materials in the positive half-cell of a Vanadium Redox Flow Battery [22], supercapacitors [23,24,25], and sensors [26]. In the fuel cell research field, CNWs have been exploited mainly as a catalyst support, replacing the classic carbon black which is easily oxidized, forming CO_2_ at the beginning and the end of the fuel cell cycles. This causes the Pt catalyst to detach and agglomerate, which, eventually, leads to performance degradation [27,28,29]. In this context, our approach is to design a method to employ CNWs, with specific MPL-tailored properties, in PEMFCs, a route not yet addressed in the literature. 

In this study, MPL layers of CNWs were directly grown on carbon paper by chemical vapor deposition assisted by a radio-frequency plasma jet (PACVD), aiming to achieve the required properties of MPLs (high hydrophobicity, good electrical conductivity, and mechanical stability) for improving the power generation of the PEMFC. By growing the CNWs directly on the carbon paper support, the disadvantages related to mechanical stability and electrical contact at the carbon paper-MPL boundary were, to a large extent, overcome. In addition, the technique also allowed us to remove the insulating Teflon binder. The nanostructures’ specific morphological and structural properties were investigated, and the thermal stability and contact angle measurements were performed on the CNW deposited on the carbon paper support. Furthermore, single-fuel-cell tests were performed on MEAs with CNWs as MPLs and compared to similar MEAs constructed with the commercially available graphene and carbon black.

## 2. Materials and Methods

### 2.1. Carbon Nanowalls Synthesis

CNW films, directly grown on the carbon paper surface (3 × 3 cm Toray Carbon Paper TGP-H-120, 5% Wet Proofing) to form the identical anode and cathode GDLs, were produced by PACVD. The deposition involved two steps, using a previously described experimental set-up [30]: (a) cleaning the carbon paper substrate surface by exposing it to a plasma bean generated in argon (1400 SCCM) mixed with hydrogen (25 SCCM) for 30 min and (b) growing the CNW film on the pretreated substrate by exposing the carbon paper to an expanding Ar plasma (1400 SCCM) injected with hydrogen and acetylene (H_2_/C_2_H_2_: 25/2 SCCM) for 60 min at 700 °C (substrate temperature). The working pressure in the chamber was about 1.3 mbar and the discharge RF power was 300 W. At the end of the deposition, the samples were cooled down freely to room temperature in an argon atmosphere. To determine the CNWs’ height and their wt.% load per cm^2^ (approx. 6 μm and, respectively, 0.3 mg cm^−2^), we also deposited carbon-nanowall layers on a silicon substrate.

### 2.2. Membrane–Electrode Assemblies 

MEAs with three different MPLs, i.e., CNWs—the material being tested—commercially available graphene (3 nm Graphene Nanopowder, Grade AQ-1, Graphene Supermarket), and carbon black (Carbon Black nanopowder >99%, particle size 13 nm)—as references—were produced based on a previously documented procedure [31]. The gas diffusion layer was composed of a 3 × 3 cm Toray Carbon Paper (TGP-H-120, 5% Wet Proofing) backing layer and a microporous layer (MPL) with 0.3 mg cm^−2^ loading for both the anode and cathode. The reference MPLs were composed of 90 wt.% carbon material and 10 wt.% PTFE (Teflon PTFE DISP 30, Chemours|DuPont™, FuelCellStore), corresponding to the minimum PTFE loading needed to balance the power performance and the water-flow management. The carbon materials and PTFE resin were dispersed in isopropanol, ultrasonicated with an ultrasonic liquid processor for 10 min at 80 W cm^−2^ of sonication intensity for ink homogenization, and uniformly spray-coated on the carbon paper backing layer. 

**Figure 1:**
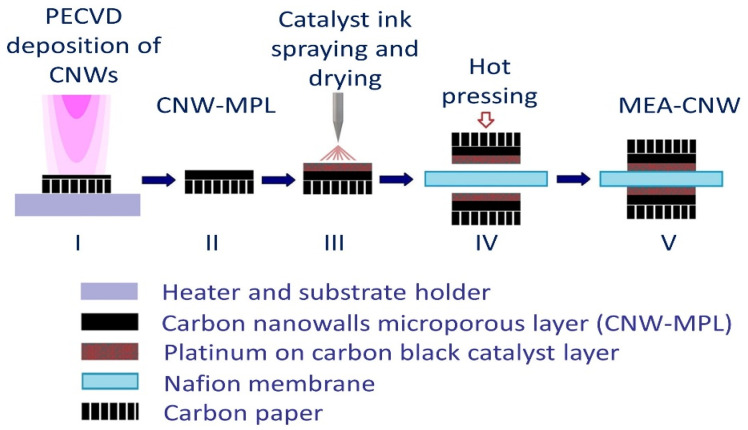
Graphic illustration of the MEA fabrication: (I) CNW deposition on the carbon paper by PECVD techniques; (II) gas diffusion layer composed of CNW as MPL and carbon paper; (III) catalyst layer deposition by spraying the platinum ink on top of the MPL (0.3 mg cm^−2^ Pt loading at the anode and 0.6 mg cm^−2^ at the cathode); (IV) hot-pressing the two catalyst-loaded GDLs against Nafion proton exchange membrane; (V) MEA_CNW ready for tests.

The catalyst layer was uniformly sprayed on the GDL surface. The catalyst consists of commercial Pt/C powder (Alfa Aesar, 60% Pt) and a 15% Nafion solution (Nafion^TM^ PFSA 5% Dispersion in alcohol-propan-1-ol and ethanol, D520, Fuel Cell Store) following a protocol described in a previous study [32]. To improve the speed of the cathodic sluggish oxygen reduction reaction, which is responsible for most of the PEMFC’s overall voltage loss, the cathode side requires a higher platinum loading than the anode, usually double or even several times greater [33]. Thus, the catalyst loadings were 0.3 mg cm^−2^ at the anodes and 0.6 mg cm^−2^ at the cathodes. Nafion perfluorinated membranes (Dupont) were used as proton exchange membranes after activation. MEAs were obtained by hot-pressing against the membranes at 125 °C and 0.4 kN cm^−2^ of pressure for 15 min. The entire process diagram is represented in Figure 1. The MEA samples having carbon black, graphene, or CNWs as MPLs are referred to as follows: MEA_Ref_CB, MEA_Ref_G, and MEA_CNW.

### 2.3. Characterization Methods

The nanostructures’ morphology was characterized using Scanning Electron Microscopy (SEM, Apreo S ThermoFisher, Waltham, MA, USA), at a working voltage of 10 kV and pressure of 3 × 10^−3^ Pa. SEM micrographs were acquired with a maximum resolution of 0.7 nm. 

Raman spectroscopy was performed from 800 to 3800 cm^−1^ on an NRS-3100 JASCO Raman Spectroscope (Oklahoma City, OK, USA) using a green laser at 532 nm.

The X-ray photoelectron spectroscopy (XPS) analysis of the CNW’s surface chemical composition was performed using an Escalab Xi^+^ system, Thermo Scientific. The survey scans were acquired using an AlKα gun, with a spot size of 900 µm, pass energy of 100.0 eV, and energy step size of 1.00 eV. For the high-resolution XPS spectra, the pass energy was set to 10.0 eV with an energy step size of 0.10 eV. The curve fitting of the C1s and O1s spectra were carried out using a Gaussian–Lorentzian peak shape and all the spectra were corrected using C1s peaks (284.5 eV) as references.

The thermal analyses were performed using thermogravimetric analysis (TGA Mettler Toledo, model Star1, Greifensee, Switzerland) at an airflow rate of 60 mL/min, at a heating rate of 10 °C min^−1^, in the temperature range of 25–1000 °C. 

Four-probe electrical measurements were performed by using a Keithley 2400 source meter and a Keithley 6517a electrometer at room temperature.

The hydrophobic character was determined according to the sessile drop method by calculating the static contact angles. The contact angle measurements were taken by placing drops of 1.5–2 μL of deionized water on the sample surface at room temperature. Temporal series of frameworks were captured by a CCD camera (40 images were captured with two different time frames: 16 ms and 1 s). The contact angle value was obtained using the Young/Laplace equation in the fitting calculation. All samples were tested after 4 days when the wettability of the samples became stable due to the aging phenomena.

MEA single-cell testing was performed on a BekkTech BT-512 single-cell-fuel-cell test station (BekkTech LLC, Loveland, CO, USA) controlled by an Agilent 6060 B system (Santa Clara, CA, USA), providing precise control over the gas temperature, pressure, humidity, and flow rates, as well as the cell temperature. The set operating conditions were as follows: 80 °C cell temperature, 40%/80% relative humidity (RH), 200 SCCM hydrogen flow rate at the anode, and 800 SCCM airflow rate at the cathode. Furthermore, a series of electrochemical experiments were carried out in the same configuration as the fuel-cell testing: cyclic voltammetry (CV) for MEA activation before single-cell testing, and in situ electrochemical active area evaluation (ECA), and linear sweep voltammetry (LSV) for hydrogen fuel crossover testing before and after single-cell testing. The experiments were performed on an OrigaFlex OGF500 potentiostat/galvanostat system (±5 nA to ±500 mA current range, ±15 V applied voltage, OrigaLys ElectroChem SAS, Rillieux-la-Pape, France) according to the standard procedures described in our previous studies [31,32].

## 3. Results and Discussions

### 3.1. Morphological Properties

**Figure 2:**
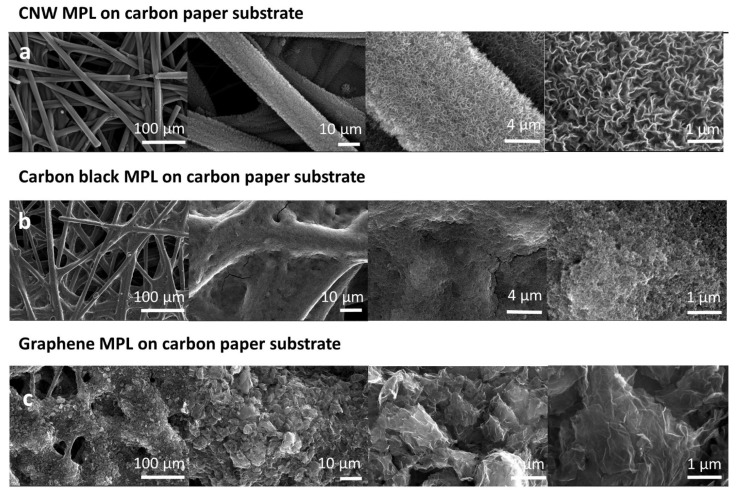
Top-view SEM images showing the MPL anchoring pattern on top of the carbon paper and the material structural details for (**a**) carbon nanowalls, (**b**) carbon black, and (**c**) graphene, at different resolutions.

The SEM investigations of the base MPL’s coating pattern presented completely different morphologies for the three materials (Figure 2). It was found that the CNWs formed the most uniform coverage, while graphene formed the roughest surface. In the top-view SEM images in Figure 2b, the carbon paper covered with carbon black presents uneven fiber coverage, with blocked pores and small cracks of less than 100 nm wide and 5 μm long. The base material is composed of similar small round particles of 10–15 nm which form a layer with extremely fine pores on top of the carbon paper.

The graphene-based microporous layer morphology was composed of wrinkled lamellar stacked flakes of 4–10 μm, oriented in the longitudinal direction (Figure 2c). This material displays a higher degree of agglomeration and compactness, as it covers a higher surface area from the carbon paper’s porous area than the carbon black due to its higher volume per cm^3^.

By using different electron microscopy resolutions, one can study the structure of CNWs in detail, identify their properties, and investigate their growth mechanism. The top-view surface images showed the CNWs’ morphology and structure growing on different substrates (carbon paper and silicon) at the nanoscale. Figure 2a presents top-view SEM images of CNWs which are directly attached to the carbon paper’s upper fibers, covering the whole top surface. They consist of randomly vertically orientated two-dimensional nanowalls, which form a slightly inter-connected network of nanographene sheets, oriented perpendicular to the substrate. The individual nanowall length is in the range of 500 nm–1 µm, while the edge thickness is less than 50 nm. 

**Figure 3:**
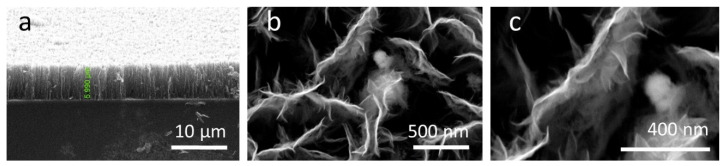
SEM images of CNW on silicon substrate: (**a**) cross-section of the CNW film showing a ~6 µm height and less than 10 nm edges width; (**b**) top-view of CNW’s wall surface details showing the hydrophobicity-enhancing patterning and the distances between individual walls and (**c**) top view of CNW’s individual wall length and structure.

The vertical form of the individual nanowalls and their height (~6 µm) can be observed in Figure 3a. This patterning was induced by the high values of working temperature and the local electrical field created by the applied power (700 °C and 300 W) which forced the carbon sheets to curl up on vertical walls [15,34]. The structure of the graphene porous network exposes an assortment of tubular open spaces which extend vertically, across the layer, from bottom to top (Figure 3a,b). This arrangement is extremely favorable to fuel-cell MPLs as it permits the unhindered passing of water and gases to the catalyst layer.

The top view of the individual wall structure (Figure 3c), with wrinkled walls, presents a similar shape to other graphitic structures produced via chemical methods [35]. The overall network structure depends on the presence of hydrogen in the discharge, as it plays an important role in the arrangement of sp^2^- and sp^3^-bonded carbon as it etches away the disordered forms of carbon [30].

### 3.2. Structural Characterization

**Figure 4:**
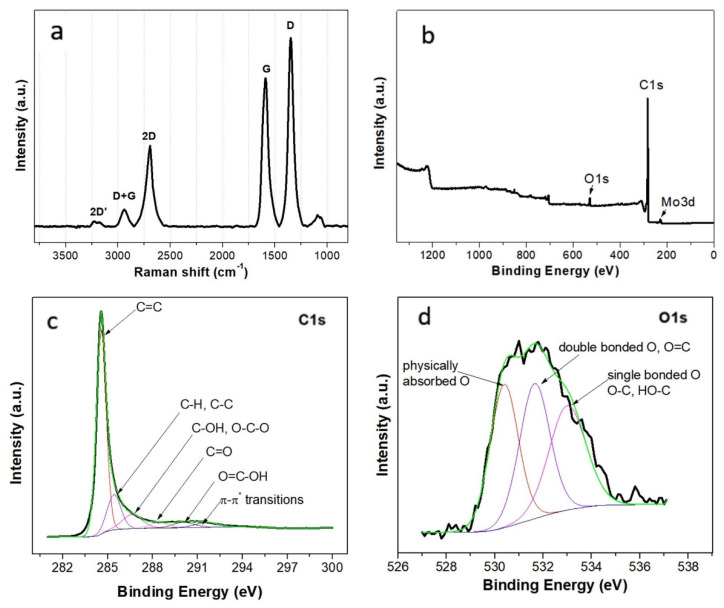
(**a**) Raman spectra of CNWs; (**b**) XPS survey mode presenting the main components of the CNWs; (**c**) primary C1s envelope and (**d**) O1s envelopes of minor constituents with their deconvoluted main components.

Raman spectroscopy is extensively used to characterize carbon-based materials as all carbon materials share a few common feature peaks which provide important information about the atom bonding, crystalline/amorphous degree, etc. A Raman spectrum of pristine CNWs with six prominent peaks is presented in Figure 4a.

The low-intensity 1100 cm^−1^ band peak corresponds to unreformed sp^3^ vibrations and is attributed to the presence of eclipsed bonds, forming fused pentagonal rings [36]. The 1350 cm^−1^ D-peak is induced by the structural point-like defects of sp^2^ carbon atoms in a ring. The G band, specific to all graphitic materials, points to the bond stretching of all pairs of sp^2^ atoms in the graphite ring structure (C–C bonds). The pick is slightly shifted to the infrared region of the electromagnetic spectrum, at 1587 cm^−1^, due to the oxidation process. The disordered degree of the structure can be determined from the ratio between the area intensities of the D and G picks. The I_D_/I_G_ peak intensity ratio, estimated at 1.27, suggests a high density of defects, arising from the amorphous carbon, or the edges of the graphene planes, vacancies, or functional groups [22].

The peak at about 2700 cm^−1^, known as second-order D-band or 2D-band demonstrates that the material contains highly oriented amorphous carbon structures. This assumption is confirmed by the presence of the D + G band (2940 cm^−1^) and 2D-band (3180 cm^−1^), which is consistent with the high electrical conductivity and the elevated electrochemical response of MEA-CNW. The I_2D_/I_G_ peak intensity ratio of 0.55 is typical for multi-layer graphene structures, in agreement with the literature [37,38]. 

The X-ray photoelectron spectroscopy of pristine CNWs (Figure 4b) presents prominent carbon C1s picks in the 283–295 eV range, low-intensity oxygen O1s in the 528–536 eV range, and small traces (less than 1%) of molybdenum Mo3d which appear due to the gas injection nozzle which is etched during plasma deposition. The material presents a small percentage of atomic oxygen (6.2%), incomparable to the high content of carbon (93.4%). However, the bounded oxygen is extremely important as it is in a great measure responsible for the high hydrophilicity of the CNWs. 

To determine the components of the C1s and O1s groups, the individual XPS spectra were fitted by Gaussian–Lorentzian functions. C1s deconvoluted scans (Figure 4c) are dominated by a preeminent C1s peak at 284.5 eV, assigned to sp^2^-hybridized C=C, followed by a long tail up to 291.5 eV, which includes a whole range of secondary-carbon-containing species. These deconvoluted picks were identified as: sp^3^ C-C and C-H at 285.5 eV, C-OH and O-C-O at 286.6 eV, C=O at 288.4 eV, O=C-OH at 290 eV, and π-π* transitions at 291.5 eV. The O1s scan (Figure 4d) indicates that oxygen is mainly found as adsorbed oxygen at 530.4 eV and as double-bonded oxygen as C=O species (aliphatic and aromatic carbonyl) at 531.7 eV. It also presents minor single-bonded oxygen species such as hydroxyl (C-OH) and C-O groups at 532.9 eV [39].

### 3.3. Thermal Analysis

**Figure 5:**
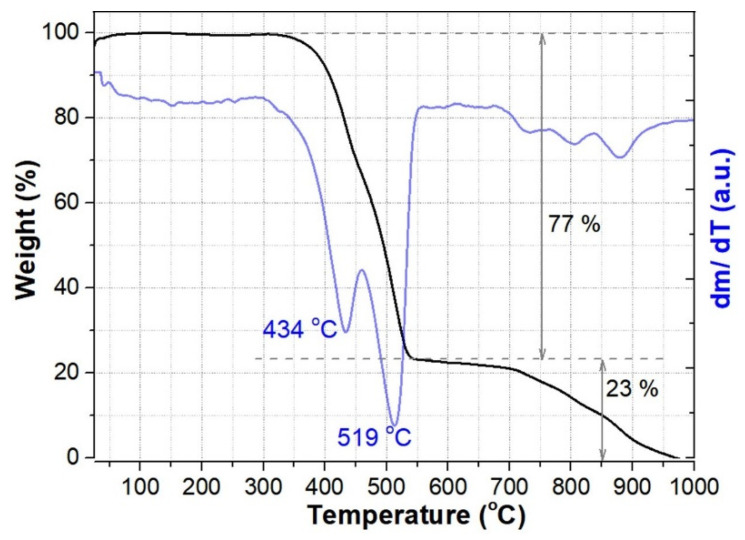
Thermogravimetric analysis of CNWs, at an airflow rate of 60 mL/min at a heating rate of 10 °C min^−1^, shows thermal stability up to 390 °C.

For a better understanding of the thermophysical and thermochemical behavior of CNWs, thermal analysis studies have been performed at an airflow rate of 60 mL/min, and at a heating rate of 10 °C min^−1^. The thermograms of the CNWs are presented in Figure 5. 

Besides the slight mass increase up to 75 °C that could be explained by the adsorption of some gas species from the atmosphere, no significant degradation occurs in air atmosphere up to 392 °C, proving the robustness and stability of CNW samples at the moderate temperatures required for fuel cell operation. Figure 5 also shows derivative thermogravimetric curves with two typical peaks attributed to the oxidation of amorphous carbon and the CNWs. The oxidation of amorphous carbon was found to have a maximum reaction rate at 434 °C, close to the other data from the literature reporting it in the interval 350–428 °C [40,41,42]. A major weight loss of about 77% is attributed to these two degradation reactions, occurring in the temperature interval of 350–550 °C. The following process, with a weight loss of about 23%, corresponds to the oxidation of polyaromatic carbon shells [43].

### 3.4. Physical Properties 

The four-probe measurements of the CNW layer presented electrical sheet resistivity values of 0.323 Ω·cm. Considering the fact that the CNW thickness determined by SEM investigations to be 5.99 µm, we calculated the film conductivity, which was 30.9 S cm^−1^, after performing the Hall Effect and resistivity measurements. All the calculations were discussed in detail in Ref. [34].

**Figure 6:**
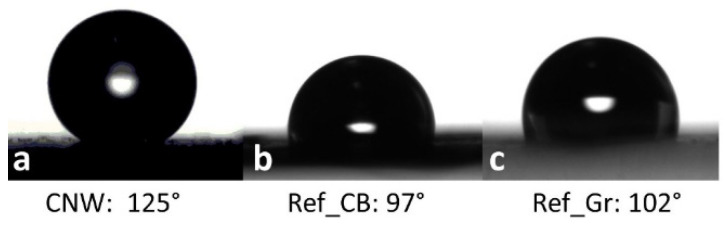
Contact angle measurements of CNW (**a**) and reference materials: carbon black (**b**), respectively, and graphenes (**c**) deposited on carbon paper.

The contact angle measurements shown in Figure 6 indicated the higher hydrophobic profile of the CNW layer, with static contact angles of about 125° in comparison with 97° for carbon black or 102° for graphene. The CNW behavior is directly related to the material topography, as the hydrophobicity increases with the decrease in defects from the carbon network. Moreover, the high concentration of oxygen is considered to be partially responsible for the high hydrophilicity of the CNWs [39]. 

### 3.5. Fuel Cell Performance Testing

**Figure 7:**
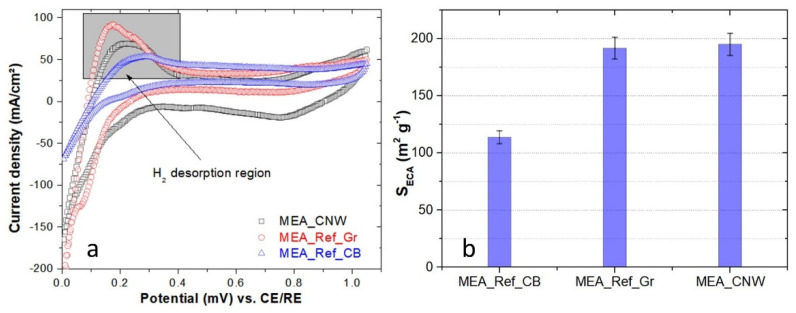
(**a**) CV tests were performed directly on MEAs, at a low scanning rate of 20 mV s^−1^ while feeding the anode (reference/auxiliary electrode) with nitrogen and the cathode (working electrode) with air, both at backpressure of 70 kPa, at 80 °C and 80% RH. (**b**) The electrochemically active surface of platinum catalyst in MEA with different MPLs: carbon black, commercial graphene, CNWs.

Before performing single-fuel-cell tests, a series of electrochemical experiments were carried out in the same configuration as the fuel-cell testing: namely, catalyst activation by cyclic voltammetry and in situ electrochemical active area evaluation. The catalyst activation process implied a large number of cycles performed at a high scanning speed, after the system has reached the set temperature and relative humidity, and ensured the efficient cleaning and restoration of the catalyst layer (Figure S1, Supplementary Information). The voltammograms showed typical features of Pt catalyst activity for all samples: the potential interval 0–0.3 V vs. CE/RE corresponds to the hydrogen adsorption/desorption region; between 0.3 V and 0.45 V no electron transfer processes occur (the double-layer region); in the range 0.5–0.65 V weak peaks appear due to oxygen species absorbed on the carbon material and, at the other end of the interval, the platinum oxide growth/stripping zone is identified (see Figure 7). 

Furthermore, experiments continued with low-scanning-rate CVs to evaluate the electrochemically active surface. The electrochemically active surface, *S_ECA_* [m^2^ g^−1^] of the platinum catalyst is evaluated directly in the single-fuel-cell testing configuration (Figure 7) using the following equation:

(1) $$S_{ECA}=\frac{Q_{H}}{c_{Pt}m_{Pt}\upsilon }$$

where *Q_H_* is the hydrogen desorption charge estimated by the integration of the area under the curve of the corresponding peak, *c_Pt_* is the Pt single-crystallite hydrogen adsorption constant, 210 µC cm^−2^, *m_Pt_* is the Pt load on the cathode side, and *υ* is the scanning rate, 20 mV s^−1^.

ECA is a measure of the activity of the catalyst layer and it is influenced by the robustness of the contact with the MPL and by the deposited layer thickness [44,45]. The tests revealed higher ECA in the case of graphene and CNWs than for carbon black, which can be attributed to a firmer contact of the catalyst layer with the MPL. Faster electron transfer to the current collectors due to the highly conductive CNWs or graphene, advanced 2D materials can also enhance ECA. Another aspect to consider is the Teflon material present in the reference MEAs that might hinder electron transfer. 

**Figure 8:**
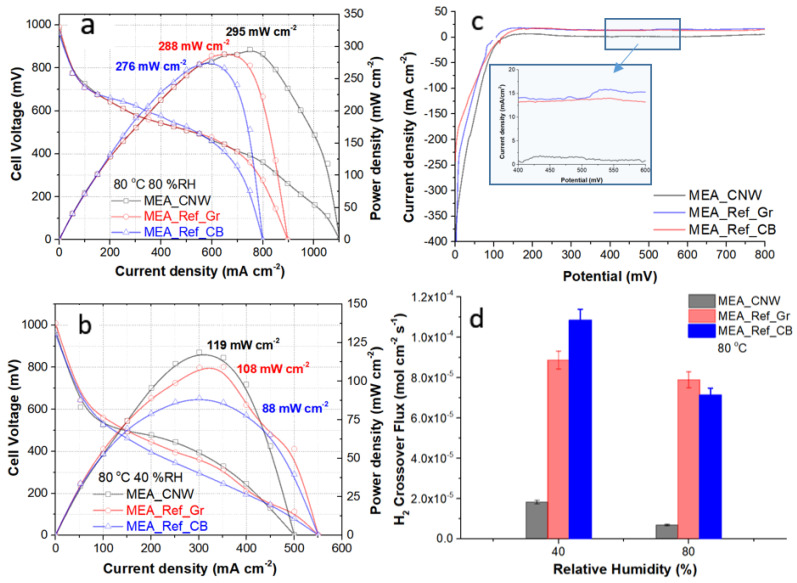
Polarization curves of comparative I–V and power density profiles between the MEA_CNW and reference MEAs, MEA_Ref_Gr and MEA_Ref_CB at 80 °C and 80% relative humidity (**a**) and at 80 °C and 40% relative humidity (**b**), respectively, with peak power densities given next to the corresponding profiles. In situ polarization testing was performed at 70 kPa of overall pressure in current sweep mode at constant flow rates of 200 SCCM of hydrogen at the anode and 800 SCCM of air at the cathode. (**c**) Hydrogen crossover testing by in situ linear sweep voltammetry for MEA_CNW, MEA_Ref_Gr, and MEA_Ref_CB at 80 °C and relative humidity of 80%; (**d**) hydrogen crossover comparative results for tested MEAs at 80 °C and relative humidities of 40% and 80%.

The polarization curves of comparative I–V and power density profiles between the MEA_CNW and the reference MEAs at 80 °C, and 80% and 40% relative humidity (Figure 8a,b) display low open-circuit losses due to low fuel crossovers, with the activation polarization overpotential not exceeding ~0.2 V below the ideal voltage. Furthermore, the ohmic polarization losses are similar, at 80% RH, while at lower relative humidity MEA_CNW, the slope of the polarization curve in the mid-section is significantly lower than in the case of both reference MEAs, i.e., lower internal resistance due to the CNWs enabling better layer contact and electron transfer at the interface GDL/catalyst. 

At the other end of the polarization curves, the maximum current density measured at 80% RH was 1100 mA cm^−1^ with 300 mA cm^−1^ over the carbon black reference. Nevertheless, at low relative humidity, the maximum current density dropped to 500 mA cm^−1^, 50 mA cm^−2^ lower than the references, due to concentration losses. The hydrophobicity of the CNW layer reduces the water discharge capability within the GDL, especially at low relative humidity [46].

In terms of peak power output, the CNW-based MEA reached 295 mW cm^−2^ at the optimal conditions of 80 °C and 80% RH, which was slightly higher than the 288 mW cm^−2^ value for the graphene reference and the 276 mW cm^−2^ value for the carbon black reference. The CNW-based MEA also outperformed the other MEAs based on different carbon materials tested in our laboratory in similar experimental conditions at 80 °C and 80% RH, such as graphene produced by the supercritical CO_2_ method (125 mW cm^−2^), and carbon xerogel (135 mW cm^−2^) [31]. On the other side, at a low relative humidity of 40%, the peak power output of the MEA with CNWs was higher by about 35% than the reference with carbon black, reaching 119 mW cm^−2^.

After taking the polarization measurements, the MEA integrity was assessed through in situ hydrogen crossover testing (Figure 8c). Linear sweep voltammetry (LSV) was performed on the cathode where any hydrogen crossing from the anode side was instantaneously oxidized under mass-transfer-limited conditions. The cathode is supplied with nitrogen while voltage is swept at a rate of 4 mV s^−1^ from 0 V to 0.8 V vs. CE/RE. The hydrogen crossover flux, *C_H_* [mol cm^−2^ s^−1^], is calculated using the formula:

(2) $$C_{H}=\frac{J_{lim}}{n\;F}$$

where *J_lim_* [mA cm^−1^] is the limiting current density, *n* = 2 is the number of exchanged electrons in the reaction and *F* is the Faraday constant.

The crossover from the anode to the cathode through the membrane is an indicator of pinhole formations in the membrane and the overall integrity of the MEA. The current density was determined by the crossover hydrogen consumption rates at the cathode. After 0.3 V, we noted a stabilization of the current due to the termination of hydrogen desorption. Comparing the LSV analyses of the samples revealed that the hydrogen crossover fluxes are lower for CNW-based MEAs than for carbon black or graphene MEAs. One reason for this could be that the uniformity of the CNW layer synthesized directly on the carbon paper ensures a smoother contact area for the catalyst layer and for the membrane during the hot-pressing MEA fabrication. Our results proved that the CNW improved resistance to hydrogen permeation, both at low and high humidity, as the membrane is subjected to less local pressure than in the case of carbon black and graphene deposited by spraying from Teflon-based solutions.

## 4. Conclusions

The inexpensive CNW thin films have been proven to be appropriate microporous layers in PEMFCs. From the deposition conditions, the specific MPL properties were tailored to present a high hydrophobic character, with good electrical conductivity. By growing the CNWs directly on the carbon paper support, to a large extent, we overcame the disadvantages related to mechanical stability and electrical contact at the carbon paper-MPL boundary.

Owing to the interconnected networks of 2D-graphene nanostructures, MEAs using CNWs exhibited improved performance in terms of high power density at both low and high relative humidity ranges when compared to carbon black and graphene. The arrangement of the graphene planes eliminates three of the issues which plague the classical microporous layer concerning mechanical stability, water management, and electrical conductivity. The mechanical stability is improved by growing stable CNW structures directly on the carbon paper substrate. The electron transfer from the catalyst to the substrate is facilitated by the deposition of graphene directly on the substrate, by their perpendicular structure and due to the elimination of the insulating binder, proven by the increased electrochemical active area of the catalyst. The issues concerning water management are also eliminated due to the high hydrophobic behavior of CNW structures and due to the vertically aligned nanowall structures which prevent water saturation and enhance the oxygen mass transport at the cathode’s reaction sites.

Single-cell-fuel-cell tests provide insights regarding the behavior of MEAs during low humidity conditions and cell flooding at low or high temperatures. CNW-based MEAs exhibit the highest peak power densities of 295 mW cm^−2^, corresponding to 500 mW cm^−2^ mg^−1^ of cathodic Pt load, with the maximum current density of 1100 mA cm^−1^, at the optimal conditions of 80 °C and 80% RH, considerably higher than the carbon black reference. These results represent a significant step forward in the development of PEMFCs, marking the CNW-based GDLs as alternatives, which offer reasonable performance at a less demanding power output, and for a lower cost. Future research needs to address possible methods for improving the performance of carbon nanowalls, rendering this alternative material even more efficient.

## Data Availability

The data presented in this study are available on request from the corresponding authors.

## Supplementary Materials

The following supporting information can be downloaded at: https://www.mdpi.com/article/10.3390/membranes12111064/s1, Figure S1: MEA activation via cyclic voltammetry.
