# Supplementary material for: Carbon-Nanowall Microporous Layers for Proton Exchange Membrane Fuel Cell
Source: Membranes (Basel). 2022 Oct 29;12(11):1064. doi: 10.3390/membranes12111064 (PMC9698599; doi:10.3390/membranes12111064)
Supplement: Supplementary file 1 [file membranes-12-01064-s001.zip › membranes-1973464-supplementary.pdf]

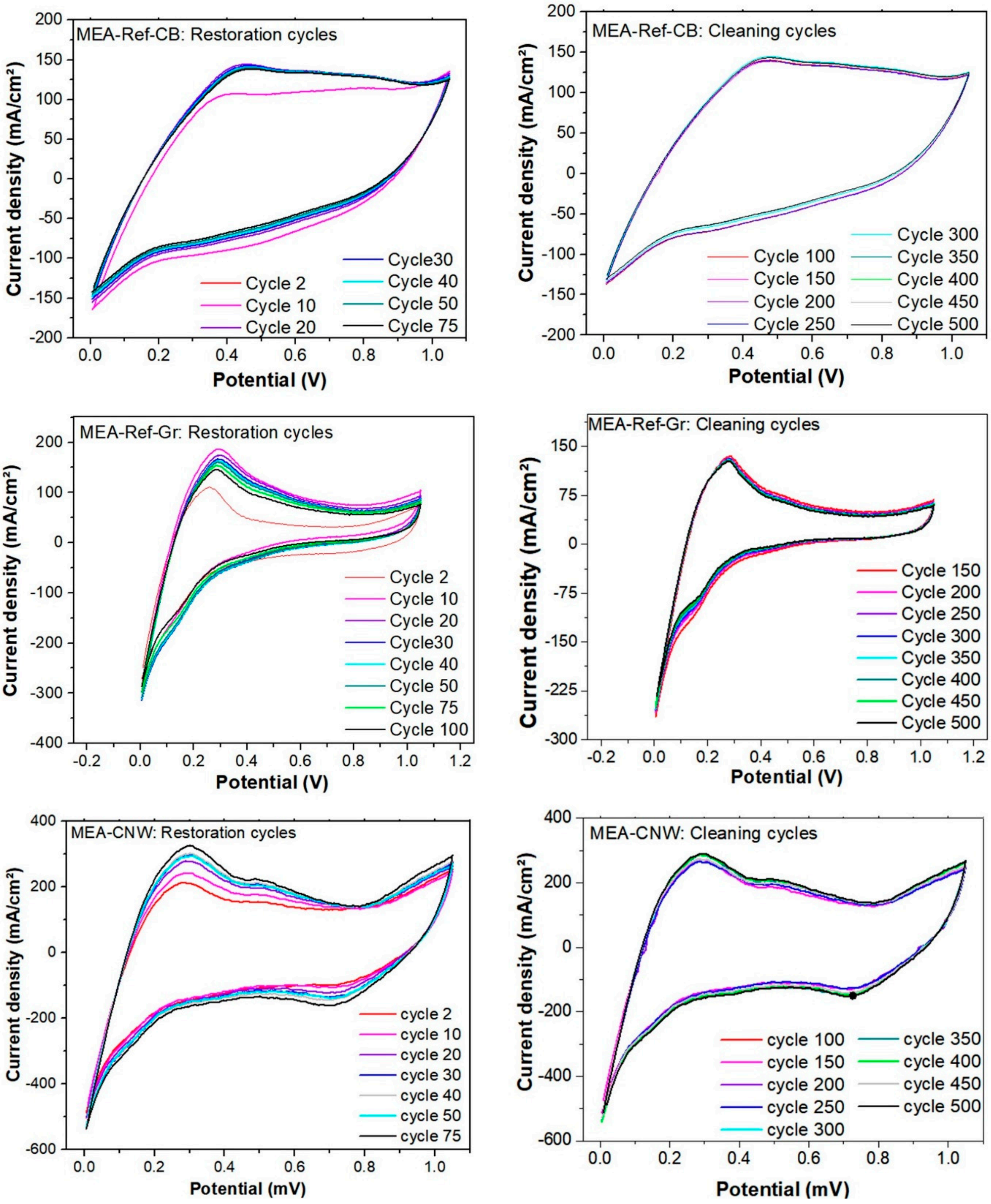

Figure S1. MEA activation via cyclic voltammetry, showing the cleaning and restoration cycles for the CNW-based MEA, and for the reference MEAs with carbon black and graphenes. The hydrogen desorption peaks are more intense in the case of CNW, thus the activation of the MEA_CNW is much faster. CVs show a selection of cycles out of a total of 500 until stabilization is achieved. MEA activation was conducted under the following stable conditions: 70 kPa backpressure pressure, 80 °C temperature and 80% RH, 150 SCCM H_2 flow at the anode and 150 SCCM N_2 flow at the cathode.
